# Genetic Diversity Analysis of Olive Germplasm (*Olea europaea* L.) With Genotyping-by-Sequencing Technology

**DOI:** 10.3389/fgene.2019.00755

**Published:** 2019-08-21

**Authors:** Shenlong Zhu, Erli Niu, Ainong Shi, Beiquan Mou

**Affiliations:** ^1^Institute of Crops and Nuclear Technology Utilization, Zhejiang Academy of Agricultural Sciences, Hangzhou, China; ^2^Department of Horticulture, University of Arkansas, Fayetteville, AR, United States; ^3^U.S. Department of Agriculture, Agricultural Research Service, Salinas, CA, United States

**Keywords:** olive, genotyping-by-sequencing technology, single-nucleotide polymorphism exploration, phylogenetic analysis, genetic diversity

## Abstract

Olive (*Olea europaea* L.) is a very important edible oil crop and has been cultivated for about 4,000 years in the Mediterranean area. Due to its nutritional and economic importance, researches on germplasm characterization received extensive attention. In this study, using the genotyping-by-sequencing (GBS) technology, we carried out genetic diversity analysis on 57 olive cultivars with different geographical origins. In total, 73,482 high-quality single-nucleotide polymorphisms (SNPs) with minor allele frequency (MAF) > 5%, call rate > 50%, and heterozygosity rate < 10% were obtained at the whole genome level. Genetic structure and phylogenetic analysis showed that the 57 olive cultivars could be classified into two groups (Group I and Group II). No clear geographical distributions of cultivars were observed generally between the two groups. The average nucleotide diversities (π) specific for Group I and Group II were 0.317 and 0.305. The fixation index (*F*_ST_) between Group I and Group II was 0.033. In Group II, cultivars could be further divided into two subgroups (Group IIa and Group IIb), which seem to be associated with their fruit sizes. The five Chinese-bred cultivars were all clustered in Group II, showing a closer genetic relationship with those from the central Mediterranean region and limited genetic background. It is therefore necessary for Chinese olive breeding programs to incorporate other genetic basis by utilizing germplasm from the other regions particularly from the east Mediterranean region as breeding parents. The results showed that GBS is an effective marker choice for cultivar characterization and genetic diversity analysis in olive and will help us better understand the genetic backgrounds of the crop.

## Introduction

Olive (*Olea europaea* L.) is one of the valuable fruit trees and the second largest woody oil plant in the world. Olive tree is native to Asia Minor and has been domesticated and cultivated around 4,000 years in Mediterranean countries (Zohary and Spiegel-Roy, 1975; Zohary and Hopf, 1994). One of the main purposes to grow olive trees is to produce fresh virgin olive oil. It is rich in monounsaturated fatty acids and has high nutritional quality, which is considered as “liquid gold” and benefits our health (Sebastiani and Busconi, 2017). Because of the high nutritional and great economic value, the olive industry has developed rapidly in recent years (Pérez-Jiménez et al., 2007; Zhan et al., 2015). At present, olives have been introduced and planted in more than 40 countries including America, Australia, and China (Kaniewski et al., 2012). Through long-term natural selection, artificial selection, cultivation, and domestication, numerous genetic resources have been formed (Wright, 1978). However, the genetic relationship among the cultivars is not yet clear, although the olive germplasms are extremely abundant. Therefore, researches on identification, classification, and genetic diversity analysis of olive cultivars are imperative, which not only helps to utilize the existing olive cultivars more effectively but also benefits genetic improvement and breeding of olive cultivars.

For these purposes, scientists have done lots of works on the germplasm resources and genetics analysis of olives. Using morphological characters, agronomic traits, biochemical markers, and molecular markers, Pontikis et al. (1980) and Ouazzani et al. (1993) elucidated 27 and 133 olive cultivars by analyzing isozyme banding patterns and applied biochemical markers to distinguish olive cultivars, respectively. Molecular markers such as random amplified polymorphism DNA (RAPD), amplified fragment length polymorphism (AFLP), sequence-related amplified polymorphism (SRAP), simple sequence repeats (SSR), inter-simple sequence repeat (ISSR), and single-nucleotide polymorphism (SNP) have been used to evaluate the genetic variation of olive germplasms (Hess et al., 2000; Rallo et al., 2000; Besnard et al., 2001; Grati-Kamoun et al., 2006; Reale et al., 2006; Isk et al., 2011; Kaya et al., 2013; Diez et al., 2015; Zhan et al., 2015; Khaleghi et al., 2017; Mousavi et al., 2017; Rienzo et al., 2018). Through the integration of molecular markers SSR, SNP and diversity array technology (DArT), and agronomical traits, Belaj et al. (2012) studied the pattern of genetic diversity among 361 olive cultivars and found that a certain association would exist between the geographical origin and genetic structure of olive, especially for the differentiated cultivars from eastern and western Mediterranean. Bartolini et al. (2005) established a public OLEA database (http://www.oleadb.it/) by integrating information of morphological, agronomical, and biochemical traits and molecular markers of more than 1,000 cultivars, which greatly benefits cultivar consultancy and further researches as well. The high efficiency and reliability of molecular markers make it an effective tool for the study of genetic diversity, molecular marker-assisted breeding, species identification, genetic map construction, and excellent gene mapping for olives.

Genotyping-by-sequencing (GBS) technology is a new method based on next-generation sequencing (Poland and Rife, 2012; Torkamaneh et al., 2016). The protocol enables high-throughput sequencing of multiplexed samples and combines genome-wide molecular marker discovery and genotyping (Torkamaneh et al., 2016). This greatly reduces the cost of gene sequencing and enables multi-sample high-throughput parallel sequencing as well. Besides, GBS technology was also available for no-reference species (Elshire et al., 2011; He et al., 2014; Torkamaneh et al., 2016). The method has been used for genetic diversity analysis in both animals and plants such as cattle (Donato et al., 2013), watermelon (Nimmakayala et al., 2014), wheat (Lin et al., 2015), spinach (Shi et al., 2017), and tetraploid ryegrass (Guo et al., 2018). İpek et al., 2016 identified 10,941 SNPs from a cross between the olive cultivars “Gemlik” and “Edincik Su” using GBS and constructed a high-density genetic map. Using the GBS data of 94 Italian olive cultivars, D’Agostino et al. (2018) obtained 22,088 and 8,088 SNPs by reference-based and reference-independent SNP calling pipeline and found the varied genetic diversity of Italian cultivars.

China is a newly emerging olive-oil-producing region in the world. It has been only 50 years since the large-scale introduction and cultivation of olive trees. It is generally estimated that the current area of olive trees in the country is about 80,000 hectares, and the annual output of olive oil is about 6,000 tons, which is simply unable to meet the demand for high-quality edible oil in the Chinese market. Most of the Chinese olive gardens have been newly built since the beginning of this century and have not yet entered fructifications or full production. Because of the huge climate and soil differences with the Mediterranean region, olive trees of most cultivars generally show a poor adaptability to local environmental conditions when grown in China, resulting in lower yield compared to their traditional cultivation regions. It is therefore important to make genetic assessments on olive germplasms so as to screen elite cultivar suitable for growing in China. Until now, the sequencing of two cultivated olive trees *O. europaea cv. Leccino* (Barghini et al., 2014) and *O. europaea* cv. *Farga* (Cruz et al., 2016) and one wild olive tree *O. europaea* var. *sylvestris* (Unver et al., 2017) has been completed. The publication and availability of genomic data provide us a quick and effective way to characterize olive germplasm resources. In this study, we analyzed the genetic background of 57 olive cultivars of different geographic origins at the whole genome level with the available database and GBS technology, aiming at carrying out the identification and evaluation of olive germplasm and providing core reference for further introduction of olive germplasm in China.

## Materials and Methods

### Plant Materials

A total of 57 olive cultivars were collected and analyzed in this study (**Table 1**), which were originally collected from eight different countries. The majority were from Italy and Spain with 27 and 19 cultivars each, while the remaining were from China (*n* = 5), France (*n* = 2), Greece (*n* = 1), Azerbaijan (*n* = 1), Portugal (*n* = 1), and Algeria (*n* = 1). *The different olive fruit sizes* (weight) were also downloaded from OLEA database (http://www.oleadb.it/) and shown in **Table 1**, including five levels: L (low: less than 2.0 g), M (medium: 2.0 to 4.0 g), MH (medium-high: 4.0 to 6.0 g), H (high: 6.0 to 8.0 g), and VH (very high: greater than 8.0 g). The Student’s *t* test was conducted to establish whether the statistics of fruit sizes were significant within different groups. Among all cultivars, 37 were used for olive oil purposes, 5 as table olive, and 15 for double purposes. All cultivars were grown in the experimental field with an average space 2 × 3 m in Zhejiang Academy of Agricultural Sciences (30^◦^18′28′′ N–120^◦^11′44′′ E), Hangzhou, Zhejiang Province, China. Young olive leaves were sampled, quickly frozen in liquid nitrogen, and stored at −70°C freezer for further analysis.

**Table 1:** Information of the 57 olive cultivars analyzed in the study.

Material no.	Olive germplasm	Origin	Fruit weight**^a^**	Usage**^b^**	Cluster/sub-cluster
1	Alfafara	Spain	H	O	Group I
2	Arbequina	Spain	L	O	Group IIa
3	Arbosana	Spain	L	O	Group IIa
4	Arroniz	Spain	M	O	Group I
5	Ascolana tenera	Italy	H	T	Group IIb
6	Bianchera	Italy	M	O	Group IIb
7	Bouteillan	France	H	T/O	Group I
8	Canino	Italy	L	O	Group IIa
9	Carrasqueno	Spain	M	T/O	Group I
10	Castellana	Spain	M	O	Group I
11	Changlot real	Spain	M	O	Group IIa
12	Chemlal de Kabylie	Algeria	M	O	Group IIa
13	Chenggu 32	China	M	T/O	Group IIa
14	Cipressino	Italy	M	O	Group IIb
15	Cobrancosa	Portugal	H	O	Group I
16	Coratina	Italy	M	O	Group IIb
17	Cornezuelo de Jaen	Spain	M	T/O	Group I
18	Cornicabra	Spain	M	O	Group I
19	Dolce agogia	Italy	M	O	Group IIa
20	Empeltre	Spain	M	O	Group IIa
21	Ezhi 8	China	M	T/O	Group IIa
22	Fecciaro	Italy	MH	O	Group IIa
23	Frantoio	Italy	M	O	Group IIb
24	Frantoio selezione quarrata	Italy	M	O	Group IIa
25	Gentile di chieti	Italy	M	O	Group IIb
26	Grignan	Italy	H	O	Group I
27	Grossa di spagna	Italy	VH	T	Group I
28	Hojiblanca	Spain	H	T/O	Group I
29	Huaou 5	China	MH	O	Group IIa
30	I-77	Italy	M	O	Group IIa
31	I-79	Italy	M	O	Group IIb
32	Koroneiki	Greece	L	O	Group IIa
33	Leccino	Italy	M	O	Group IIa
34	Limona	Italy	M	T	Group IIa
35	Manzanilla	Spain	H	T/O	Group I
36	Manzanilla cacerena	Spain	H	T/O	Group I
37	Manzanilla sevillana	Spain	H	T	Group I
38	Maurino	Italy	L	O	Group IIa
39	Moraiolo	Italy	L	O	Group IIa
40	Morcona	Italy	M	O	Group IIb
41	Nevadillo fino	Spain	M	O	Group I
42	Nikitskii I	Azerbaijan	MH	T/O	Group IIa
43	Nociara	Italy	M	T/O	Group IIb
44	Nostrale di rigali	Italy	M	O	Group IIb
45	Olivo de caniles	Spain	–	UN	Group I
46	Pendolino	Italy	L	O	Group IIa
47	Peranzana	Italy	M	T/O	Group I
48	Picholine	France	M	T/O	Group IIb
49	Picual	Spain	M	O	Group I
50	Redondilla	Spain	MH	T/O	Group I
51	Rosciola	Italy	L	O	Group IIa
52	Santa caterina	Italy	H	T	Group I
53	Taggiasca	Italy	M	O	Group IIb
54	Verdial de badajoz	Spain	VH	O	Group I
55	Yuntai	China	M	T/O	Group IIa
56	Zen	Italy	L	O	Group IIa
57	Zhonglan	China	L	O	Group IIa

a Fruit weight: low, L (less than 2.0 g; medium, M (2.0 to 4.0 g); medium-high, MH (4.0 to 6.0 g); high, H (6.0 to 8.0 g); very high, VH (greater than 8.0 g)

b T, Table olive; O, Olive oil; T/O, Double purpose.

### DNA Extraction and GBS Library Construction

Genomic DNA of the 57 olive cultivars was extracted with the cetyl-trimethyl-ammonium-bromide (CTAB) method as described by Murray and Thompson (1980). Qualified DNA samples, after checking on agarose gel, were digested with ApeKI (New England Biolabs, USA) and then ligated to either barcoded adaptors or common adaptors. Only short samples featuring both barcode and common adaptor were enriched by PCR amplification and then purified by magnetic beads with a range of 250–300 bp. Finally, paired-end sequencing was performed on an Illumina HiSeq 2000 platform at Beijing Genomics Institute (BGI) in Hong Kong.

### GBS-SNP Procedure

The bioinformatics pipeline for GBS-SNP is summarized in **Supplementary Figure S1**. In detail, raw reads were filtered and split into clean reads by the following steps: 1) remove reads with adaptors; 2) remove low-quality reads, of which more than 50% had quality value ≤ 12; 3) remove reads whose unknown bases were ≥ 3%; 4) remove reads that do not contain barcode (4–8 bp) at 5-most of reads used to be identified by different samples (one barcode corresponds to one sample); and 5) trim the barcode after step 4 and then remove reads lacking key sequence of the enzymes at 5-most.

Clean reads were then aligned to the olive reference sequences *O. europaea* cv. *Farga* (Cruz et al., 2016) using SOAP2 software (Hurgobin, 2016). Subsequently, SOAPsnp was used to call SNP (Li et al., 2008; https://sourceforge.net/projects/soapsnp/). The main parameters are shown in **Supplementary Table S1**.

The Bayesian model was applied to calculate the probability of genotypes. The genotype with the highest probability was selected as the genotype of the sequencing individual at the specific locus. Using the consensus sequence, polymorphic loci against the reference sequence were selected and then filtered under certain requirements. The call frequency, minor allele frequency (MAF), heterozygosity rate, and polymorphism information content (PIC) (Botstein et al., 1980) were calculated and analyzed using EXCEL 2013 software based on the SNP genotyping.

### Population Characteristics and Linkage Disequilibrium Analysis

To reflect the genetic relationship of olive cultivars, the SNPs with missing data > 0.5 were excluded and the remaining data with MAF > 5% and heterozygosity rate < 10% were selected for further analysis. Genetic structure analysis was conducted using admixture 1.3 (Alexander et al., 2009) and the number of populations (*K*) was calculated from *K* = 1 to 10. Meanwhile, a phylogenetic tree was constructed using MEGA X software with the neighbor-joining method (www.megasoftware.net; Kumar et al., 2018) and further edited by Figtree software (https://sourceforge.net/projects/figtree/). The parameters were as follows: Test of phylogeny, bootstrap method; no. of bootstrap replications, 1,000; Model/method, maximum composite likelihood; Substitutions to include, d: Transitions + Transversions; Gaps/missing data treatment, pairwise deletion. Principal component analysis (PCA) was performed using TASSEL 5.0 software (https://tassel.bitbucket.io/) with an identity-by-state (IBS) matrix data. Pairwise IBS allele-sharing was calculated using PLINK V1.90 presented by multidimensional scaling (MDS) plot (Purcell et al., 2007). The correlation coefficient (*r* ^2^) of alleles was calculated to measure linkage disequilibrium (LD) in each group level using PLINK V1.90 (Purcell et al., 2007).

### Population Diversity Analysis

VCFtools (https://vcftools.github.io/) was employed to calculate the parameters of population genetic diversity. The degree of polymorphism within a population was measured by the average number of nucleotide differences per site (π; Nei and Li, 1979), and the genetic differentiation between groups was measured by fixation index (*F*_ST_; Holsinger and Weir, 2009).

## Results

### General Characteristics of GBS in Olive

To understand the genetic relationship of olive germplasm, 57 olive cultivars mainly from Italy and Spain were sequenced using GBS technology (**Table 1**). The data were presented in **Supplementary Table S2**. After filtering, raw reads were split into clean reads and finally generated 352.93 million (M) clean reads with average 6.19 M reads per sample (ranging from 3.66 M to 12.01 M). Statistics on sequence data further showed that the quality value 20 (Q20) ≥ 97.3%, quality value 30 (Q30) ≥ 92.8%, and the GC contents distributed in a range of 46.4–56.4%, indicating that GBS was a valuable molecular method qualified for germplasm characterization in olive.

### GBS-SNP Analysis

**Figure 1:**
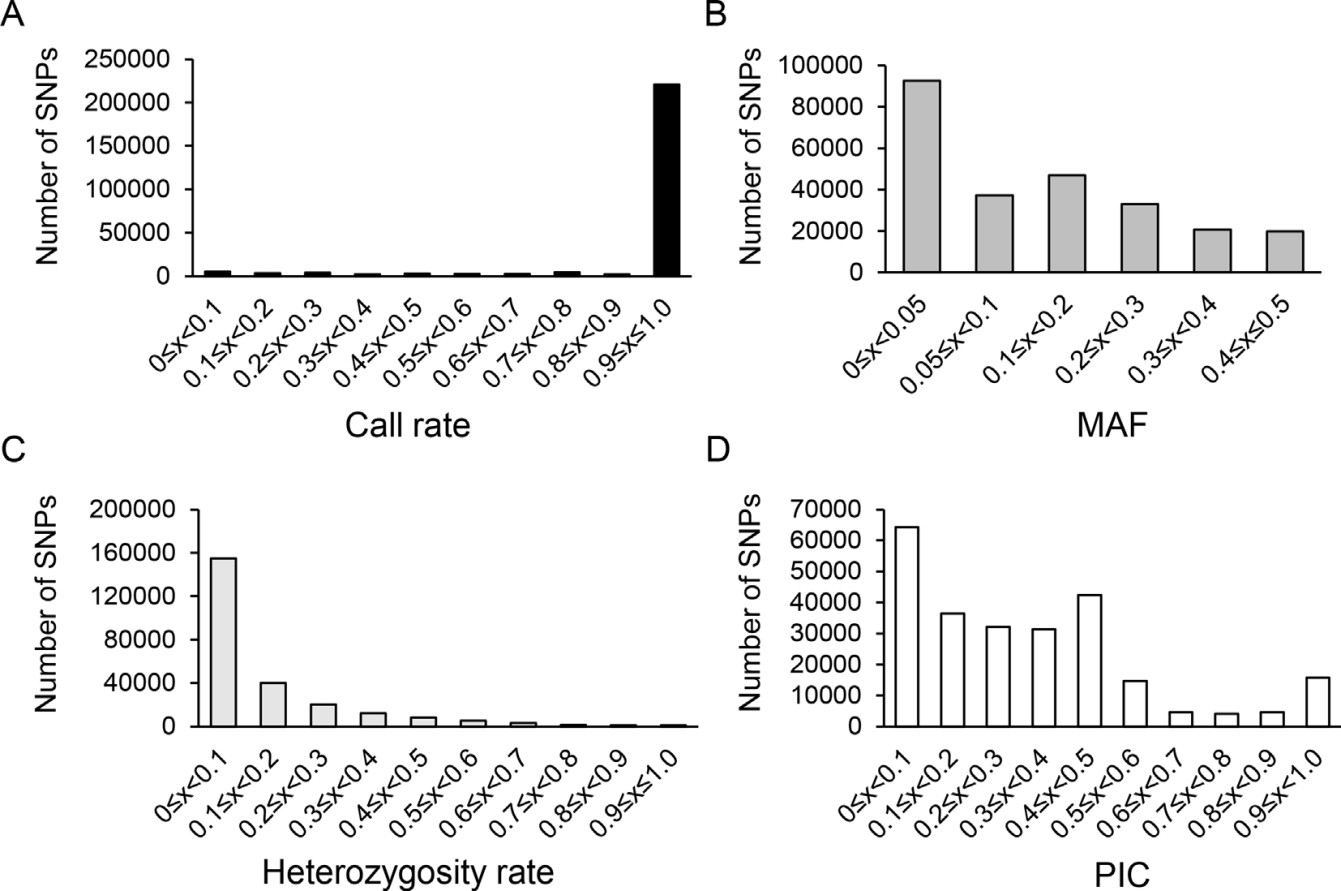
Characteristic statistics of SNPs using 250,583 SNPs. **(A)** SNPs distribution on the olive scaffolds. **(B)** Distribution of genic and inter-genic regions of selected SNPs. The *X*-axis represented the statistical SNP characteristic parameters including loci call frequency **(A)**, minor allele frequency (MAF) **(B)**, heterozygosity rate **(C)** and polymorphism information content, PIC **(D)**. The *Y*-axis represented the number of SNPs.

Clean reads were mapped to olive reference genome *O. europaea* cv. *Farga* using SOAP2 (Cruz et al., 2016; Hurgobin, 2016) and SNP call (Li et al., 2008; https://sourceforge.net/projects/soapsnp/). A total of 250,583 SNPs was generated with an average mapping rate of 44.2%. As shown in **Figure 1**, 88.0% of all the SNPs had call rate in the range of 90–100%, and 67.1% had MAF > 5%. Besides, the heterozygosity rate was mainly in the range of 0–10%, which accounted for 61.8% of all SNPs. PIC was mainly in the range of 0–50%, with 3.8% of all SNPs having a PIC = 50%.

The 250,583 SNPs were further filtered under the condition of MAF > 5%, call rate > 50%, and heterozygosity rate < 10% and generated 73,482 SNPs used for genetic diversity analysis with a mean depth of 49.5 reads/SNP. The missing calls of filtered SNPs were in the range of 0.2–3.3% with an average of 1.4%, while the heterozygous calls were in the range of 1.3–6.4% with an average of 2.9%. Various SNP types were determined as follows: [A/G] SNP type had 20,456 SNPs (27.84%); [C/T], 20,418 (27.79%); [A/C], 8,194 (11.15%); [A/T], 8,108 (11.03%); [G/T], 7,700 (10.48%); [C/G], 5,700 (7.76%). The remaining SNPs (2,900; 3.95%) displayed three or four polymorphic types. Meanwhile, among all the cultivars investigated, 13 cultivars (Bouteillan, Coratina, Ezhi 8, Hojiblanca, Huaou 5, Manzanilla, Nevadillofino, Nikitskii I, Olivo de caniles, Pendolino, Picual, Santa caterina, and Zhonglan) showed heterozygous calls of less than 2.0%, whereas 5 cultivars (Chenggu 32, Cipressino, Nociara, Nostrale di rigali, and Taggiasca) displayed higher heterozygous calls of more than 5.0%. The filtered SNPs among single cultivar are listed in **Supplementary Table S3**.

### Genetic Structure and Phylogenetic Analysis

**Figure 2:**
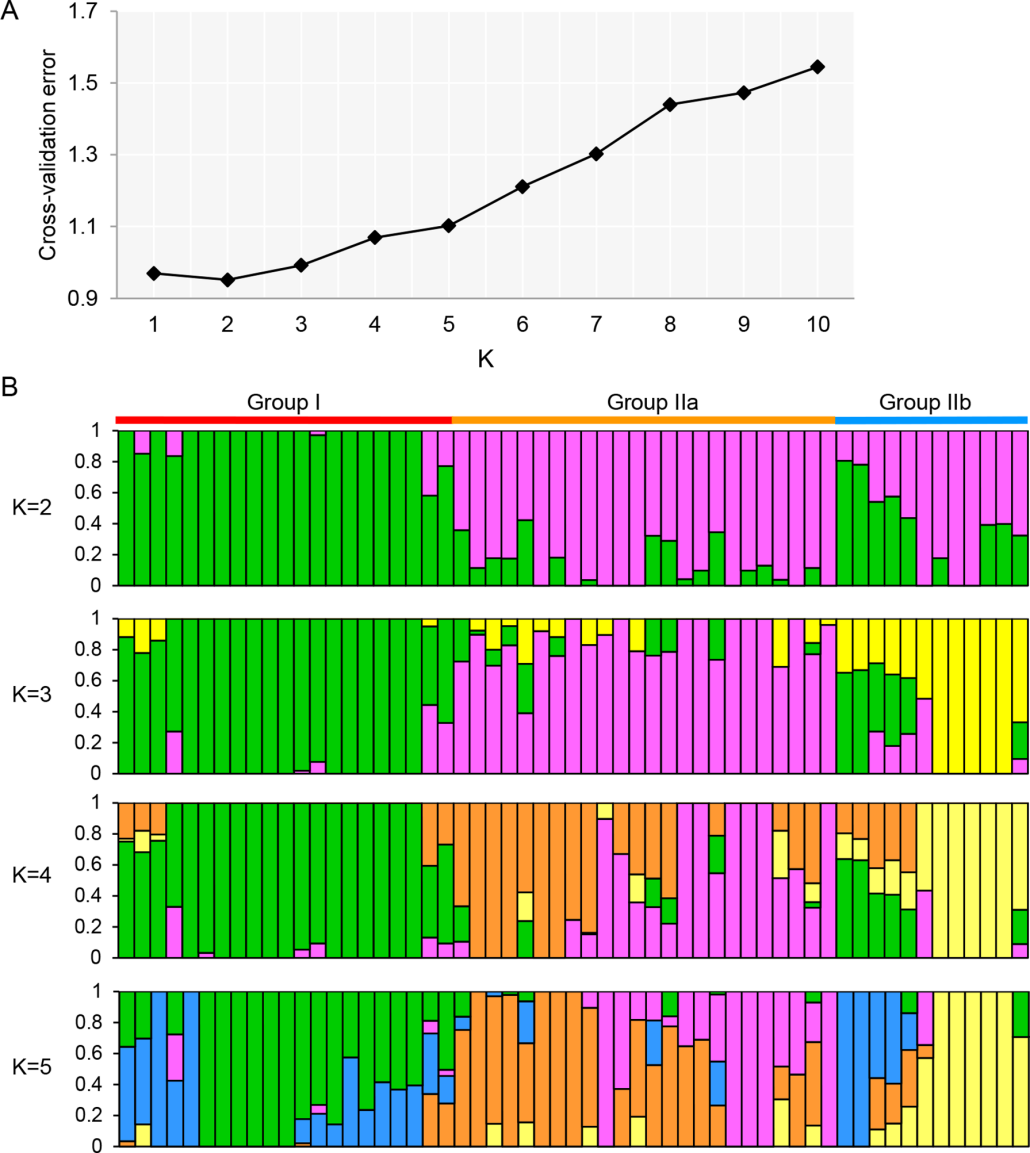
Population structure analyses of 57 olive cultivars based on the GBS-SNP genotyping. **(A)** Cross-validation plot for the number of population (K) values. The *X*-axis and *Y*-axis represented the different K values (*K* = 1–10) and cross-validation error. **(B)** Stacked bar plot for the K value = 2, 3, 4, and 5. The population structure analysis was performed by admixture 1.3 (Alexander et al., 2009). The *X*-axis represented the individual cultivar with K colored segments and the *Y*-axis represented the ancestry qi proportion, correspondingly.

Genetic structure and phylogenetic analysis were further performed to gain an insight into the genetic diversity of olive cultivars. The 73,482 SNPs of high-quality data were used to investigate the population structure among 57 olive cultivars. Using admixture 1.3, the cross-validation errors were examined under the models with *K* = 1–10. As suggested, a good value of K will exhibit the lowest cross-validation error compared to other *K values* (Alexander et al., 2009). Here, the minimum value of the cross-validation errors was 0.95 when *K* = 2 and the values continuously increased with *K* from 3 to 10 (**Figure 2A**). To classify groups, we considered a genotype unequivocally assigned to a group when its admixture coefficient was >80% (*Q* > 0.8) as previously described (Diez et al., 2015). The cultivars were classified into two groups at *K = 2, except for* 14 cultivars that could not be unequivocally assigned to any of the two groups (**Figure 2B**). The first group contained 20 cultivars from six countries (Italy, Spain, Greece, China, Azerbaijan, and Algeria), and the second group contained 23 cultivars from four countries (Italy, Spain, France, and Portugal). To further investigate the population structure, the analyses at *K* = 3–5 were also performed (**Figure 2B**). When *K* = 3, three groups were identified with 36 cultivars including a new group that consisted of 5 cultivars (Nostrale di rigali, Taggiasca, Frantoio, I-79, and Ascolana tenera from Italy). The new groups were also identified at *K* = 4 and 5. However, just 33 and 28 cultivars could be unequivocally assigned to groups, respectively.

**Figure 3:**
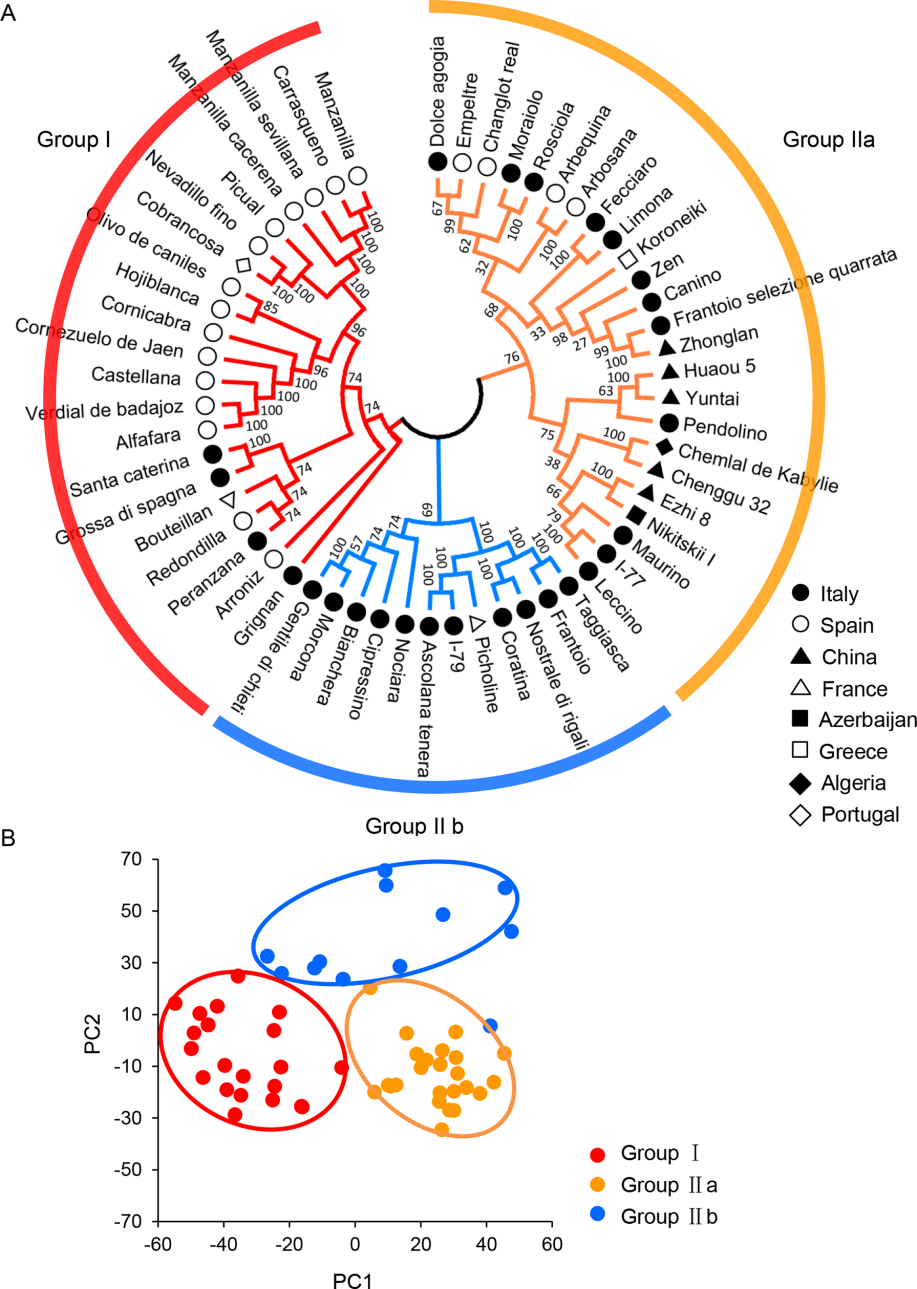
Phylogenetic analyses of olive cultivars. **(A)** Phylogenetic analysis of olive cultivars. Phylogenetic tree was constructed using MEGA X software (www.megasoftware.net) with the neighbor-joining method (Kumar et al., 2018). The parameters were as follows: Test of phylogeny, bootstrap method; no. of bootstrap replications, 1,000; Model/method, maximum composite likelihood; Substitutions to include, d: Transitions + Transversions; Gaps/missing data treatment, pairwise deletion. **(B)** Principal component analysis (PCA) of olive cultivars using TASSEL 5.0 software (https://tassel.bitbucket.io/).

With phylogenetic analysis, neighbor-joining tree using MEGA X software also clearly clustered the 57 cultivars into two main groups (Group I and Group II), which was consistent with the model-based population structure at *K* = 2 (**Figure 2B** and **Figure 3A**). Group I consisted of 21 cultivars (36.8%) from four countries, i.e., Spain (*n* = 15), Italy (*n* = 4), France (*n* = 1), and Portugal (*n* = 1), while Group II included the remaining 36 cultivars (63.2%). Group II could be further classified into two subgroups named Group IIa and Group IIb. In Group IIa, there were 24 cultivars (42.1%) from six countries, including 12 cultivars from Italy, 4 from Spain, 5 from China, 1 from Azerbaijan, 1 from Greece, and 1 from Algeria. In Group IIb, there were totally 12 cultivars (21.1%) from only two countries including 11 cultivars from Italy and 1 from France. Moreover, the distribution of the two dimensions generated by PCA of all 57 cultivars agreed well with the classification of all the cultivars into two clusters (**Figure 3B**), which was also consistent with the model-based population structure and phylogenetic analysis. The relationships among the 57 olive cultivars were further analyzed with the IBS allele-sharing values. The bin for all the cultivars filled between 0.59 and 0.88, with the majority (1,515, 94.7%) distributed in 0.65–0.75 (**Supplementary Figure S2A**). The 10 pairs with allele-sharing values > 0.85 could be seen in **Supplementary Table S4**. Besides, the multidimensional scaling (MDS) plot of genome-wide IBS pairwise distances also displayed a clear separation of two groups (Group I and Group II), while the cultivars in Group IIa and Group IIb were interlaced partially (**Supplementary Figure S2B**).

**Figure 4:**
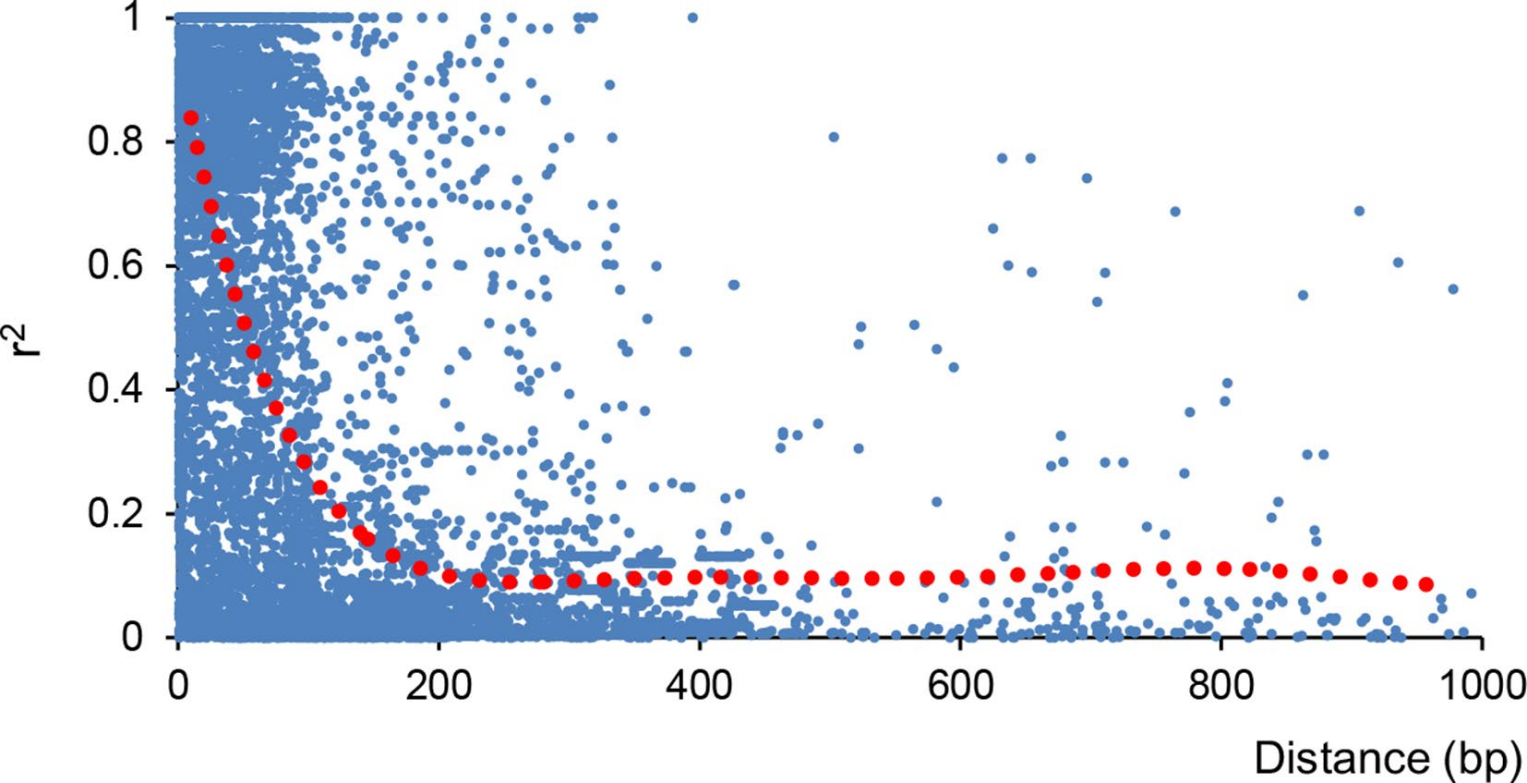
Decay of linkage disequilibrium (LD) in all 57 olive cultivars. Decay of LD indicated by correlation coefficient (r^2^) was calculated using PLINK V1.90 (Purcell et al., 2007).

Moreover, linkage disequilibrium (LD) decreased with physical distance among SNPs in all 57 olive cultivars. For more than 5,000 scaffolds that differ in size, LD decay was estimated considering only those SNP markers identified in the 30 longest scaffolds as the method described by D’Agostino et al. (2018). The extent of LD was measured as the scaffold distance when LD decreased to half of its maximum value. We also found a rapid decay of LD (**Figure 4**), with average *r* ^2^ dropping from 0.74 to 0.41 (80 bp) and 0.33 (90 bp), which was slightly higher than that in a previous report (D’Agostino et al., 2018).

### Genetic Diversity Analysis

**Figure 5:**
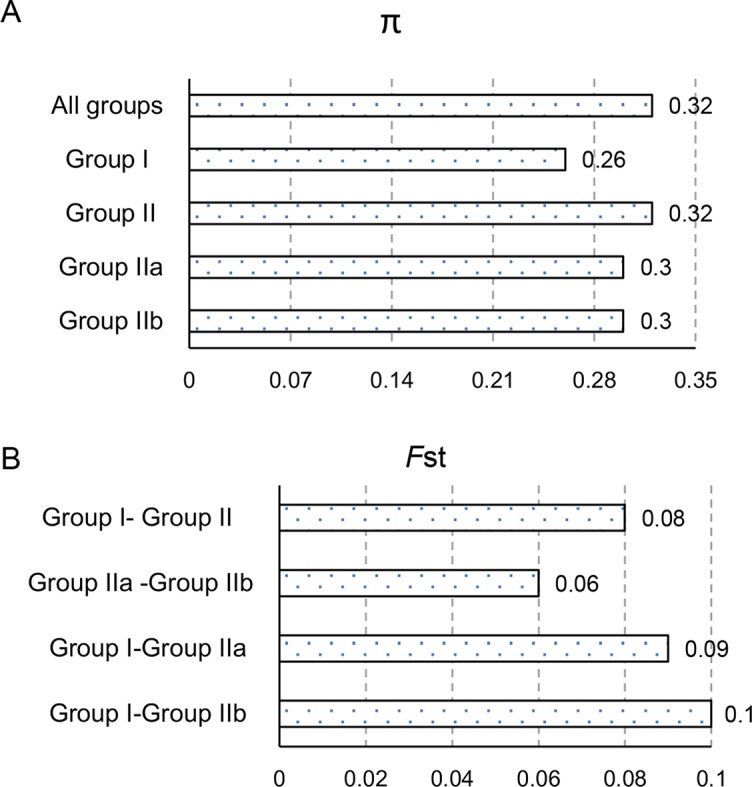
Genetic diversity analyses of different olive groups. The levels of nucleotide diversity, π **(A)** and fixation index, *F*_ST_ **(B)** between groups were calculated using VCFtools (https://vcftools.github.io/).

To explore the genetic differentiation among populations, we used VCFtools (https://vcftools.github.io/) to conduct genetic diversity analysis (**Figure 5**). The average nucleotide diversity (π) of the total sites was 0.318 for the whole set of olive cultivars, which was bigger than the π values specific for Group I (0.26) and Group II (0.32). Moreover, both of the cultivars in Group IIa and Group IIb shared the π values 0.30 (**Figure 5A**). The fixation index (*F*_ST_) for Group I–Group II, Group I–Group IIa, Group I–Group IIb, and Group IIa–Group IIb were 0.08, 0.09, 0.10, and 0.06, respectively (**Figure 5B**), indicating that the olive cultivars here displayed moderate genetic differentiation. While the π values of cultivars from Italy and Spain were 0.32 and 0.28, the *F*_ST_ of cultivars between Italy and Spain was 0.046 (**Figure 5**), which suggests that the cultivars between Italy and Spain showed a slight genetic differentiation and the cultivars from Italy had more variability.

## Discussion

### GBS-SNP Exploration in Olive

Molecular markers such as RAPD, AFLP, and SSR have been widely used in germplasm characterizations and genetic diversity analysis in plants including olive in the past two decades (Sebastiani and Busconi, 2017). In recent years, the next-generation sequencing and transcriptomic analysis become the main approaches to study the genetic characteristics of plants (Metzker, 2010; Bolger et al., 2014), due to their high efficiency of genome-wide sequencing. GBS technology, as one of the easily handled and powerful new methods, has been widely used in modern molecular breeding. To our knowledge, only a few publications related to sequencing on several olive genotypes such as *O. europaea* cv. *Leccino, O. europaea* cv. *Farga*, and *O. europaea* var. sylvestris are available (Barghini et al., 2014; Cruz et al., 2016; Unver et al., 2017), and the excavation of polymorphic loci at the whole genome only were done by İpek et al. (2016) and D’Agostino et al. (2018) with the GBS technology. In the present study, we analyzed the genetic variability of 57 olive cultivars by GBS-SNPs. A total of 352.93 million clean reads with an average data size of 588.63 Mb were generated, and as many as 73,482 high-quality SNPs were obtained subsequently after mapping to olive reference genome *O. europaea* cv. *Farga* and filtering. Compared with the *GBS*-SNP results in previous studies (İpek et al., 2016; D’Agostino et al., 2018), this study collected various cultivars with different genetic backgrounds and captured more SNPs, with the average density being higher. The genetic diversity analysis in olive germplasm is usually performed using SSR/AFLP markers and the identification of SNPs at the whole genome level is also lacking. The results will enrich the availability of genome information of olive and could be further used for genetic diversity study and modern molecular breeding.

### Genetic Diversity Among Olive Cultivars

In the previous studies, olive cultivars were classified as three gene pools including east, central, and west Mediterranean regions based on their geographic origins (Sarri et al., 2006; Belaj et al., 2012). The geographic origins had a certain correlation with the genetic differentiation (Belaj et al., 2012). The present study using model-based population analysis classified 57 cultivars into two groups (Group I and Group II), and Group II was further divided into two subgroups (Group IIa and Group IIb). Both neighbor-joining tree (**Figure 3A**) and PCA (**Figure 3B**) showed consistent results and support each other, but did not support the two groups related to geographic origins. Interestingly, based on the standard of olive fruit sizes (weight) conducted by the OLEA database (http://www.oleadb.it/), the different olive fruit sizes (weight) with five levels were observed among different groups (**Table 1**) as L (low: less than 2.0 g), M (medium: 2.0 to 4.0 g), MH (medium-high: 4.0 to 6.0 g), H (high: 6.0 to 8.0 g), and VH (very high: greater than 8.0 g). In Group I, all cultivars had medium to very high fruits, which were significantly higher (heavier) in Group I than in Group II (IIa and IIb) (*P* = 2.6 × 10−5; Student’s *t* test). In Group IIa, all cultivars had low to medium–large fruits, while in Group IIb, all cultivars except Ascolana tenera from Italy had medium fruits. The fruit size in Group IIb was bigger than that in Group IIa, with *P* = 0.045 (Student’s *t* test). The three groups had a significant association with fruit size, which was similar to the results reported by Biton et al. (2015). A set of 145,974 SNPs were developed using next-generation sequencing technology and subsequently used a subset of 138 SNPs to analyze 119 cultivars maintained in the Israeli germplasm collection (Biton et al., 2015). Comprehensive analysis showed that olive cultivars were grouped more in terms of their functions (oil, table or double purpose) than in terms of their geographic origin (Biton et al., 2015).

LD analysis in all 57 olive cultivars indicated that olive genomes had short LD distance and rapid LD decays (**Figure 4**). The LD decay distance (∼85 bp) was much shorter than that reported in pear (211 bp; Wu et al., 2018) and apple (161 bp; Duan et al., 2017). The *F*_ST_ between each group pairs (**Figure 5**) had a similar result with previous reports by D’Agostino et al. (2018) and Rienzo et al. (2018), but it was relatively lower than that in other tree plants, such as pear and apple (Duan et al., 2017; Wu et al., 2018). The above results implied a relatively weak selection and a moderate differentiation during the genetic domestication of olive, which might be due to the vegetative propagation approach and the low self-fruitful rate (Xu, 2001). Furthermore, previous studies showed that there was relative differentiation among Spanish and Italian cultivars and a clear distinction between Spanish cultivars and those from Greece and Turkey (Besnard et al., 2001; Owen et al., 2005). The cultivars in this study from Italy and Spain were distributed in both Group I and II. However, there was a clear distinction between the cultivars from the two countries within both groups; for example, none of 12 cultivars in Group IIb was from Spain. Combined with cluster analysis (**Figure 3**) and nucleotide diversity analysis, it could be inferred that compared to Spain cultivars, the Italian cultivars may have more genetic variability, which was consistent with the previous results obtained by D’Agostino et al. (2018).

As a new olive production area, most of the cultivars widely cultivated currently in China were introduced from Mediterranean countries, and some were selected and bred by Chinese olive breeding programs from cultivars such as Coligno, Ascolano Tenera, Nikitskii I, Nikitskii II, Leccino, and Kalinio (Xu, 2001; Li, 2010). Among the five cultivars developed in China in this study, Chenggu 32, Zhonglan, Yuntai, Ezhi 8, and Huaou 5, except Huaou 5 with unknown parents, the female parents of the other four cultivars were all from the central Mediterranean countries (Xu, 2001). The results of cluster analysis indicated that the five cultivars were all in Group IIa with a close genetic relationship with the cultivars from the central Mediterranean region. Previous studies showed that there was a relatively narrow genetic basis of the Chinese-bred cultivars (Xu, 2001; Li, 2010; Zhan et al., 2015). Most olive cultivars introduced in China came from the central and western regions of the Mediterranean during the 1960s to 1970s, and the germplasm from the eastern region was less (Xu, 2001). Therefore, it is necessary to introduce olive germplasm from the eastern regions of the Mediterranean in the future in order to broaden the genetic basis of the Chinese olive germplasm.

### Effectiveness of GBS for Characterizing Genetic Relationships Among Olive Cultivars

Among the cultivars analyzed in this study, Frantoio and Taggiasca, Picual and Nevadillo fino, and three Manzanilla cultivars (Manzanilla, Manzanilla cacerena, and Manzanilla sevillana) and Carrasquena were generally considered to be synonymous, which were very similar in morphological and genetic characteristics (Bartolini et al., 2005; Belaj et al., 2012). Cluster analysis showed that these cultivars did have high genetic homogeneity and clustered pairwisely or together, respectively (**Figure 3**), with higher IBS values > 0.85 (**Supplementary Table S4**). Interestingly, the cultivar Manzanilla cacerena with the other three cultivars shared IBS values of about 0.76–0.77, which were relatively low than those found in other pairs. Ezhi8 was an excellent cultivar selected from a hybrid population of free pollination. We do not know exactly its parents, but it is commonly believed that it was derived from Nikitskii I, a cultivar originated in Azerbaijan, according to their similarities in morphological traits. In this study, the two cultivars Ezhi8 and Nikitskii I were clustered together to show their close kinship with the IBS value = 0.84, confirming the general knowledge about their genetic relationships. A similar result was also found in cultivars Huaou 5 and Yuntai, which shared the highest IBS value in this study (0.88) (**Supplementary Table S4**). The two cultivars with similar morphological traits such as tree shape, leaf shape, leaf size, fruit shape, and fruit size were clustered closely as well. In summary, GBS-SNP loci here will correct effectively the relationship among different cultivars and further benefit the development of core germplasm loci.

## Author Contributions

SZ and AS conceived the study and EN carried out the analysis and wrote the manuscript. SZ, AS, and BM revised the manuscript. All authors approved the final manuscript.

## Funding

This study was financially supported by the International Science & Technology Cooperation Program of China (No. 2013DFG32780).

## Conflict of Interest Statement

The authors declare that the research was conducted in the absence of any commercial or financial relationships that could be construed as a potential conflict of interest.

## Supplementary Material

Figure S1 Bioinformatics analysis pipeline for GBS-SNP. The raw reads were subjected to quality control and split into clean reads. Using SOAP2 (Hurgobin, 2016) and SOAPsnp (Li et al., 2008), the clean reads were aligned to the olive reference sequences O. europaea cv. Farga (Cruz et al., 2016) and further to call SNP respectively. The main parameters were shown in **Supplementary Table S2**.

Figure S2 Multi-dimensional scaling (MDS) plot of identity-by-state (IBS) distance matrix. Pair-wise IBS allele-sharing using the 73,482 SNPs among 57 olive cultivars were calculated using PLINK96 V1.90 and visualized with the MDS plot (Purcell et al., 2007).

Table S1 The main parameters for sequences alignment and SNP calling.

Table S2 Summary of sequencing data of 57 olive cultivars.

Table S3 Statistics of GBS SNP genotyping.

Table S4 Pairs of cultivars with identity-by-state (IBS) allele-sharing values > 0.85.
