# Supplementary figures and images for: Genetic Diversity Analysis of Olive Germplasm (Olea europaea L.) With Genotyping-by-Sequencing Technology
Source: Front Genet. 2019 Aug 21;10:755. doi: 10.3389/fgene.2019.00755 (PMC6712157; doi:10.3389/fgene.2019.00755)

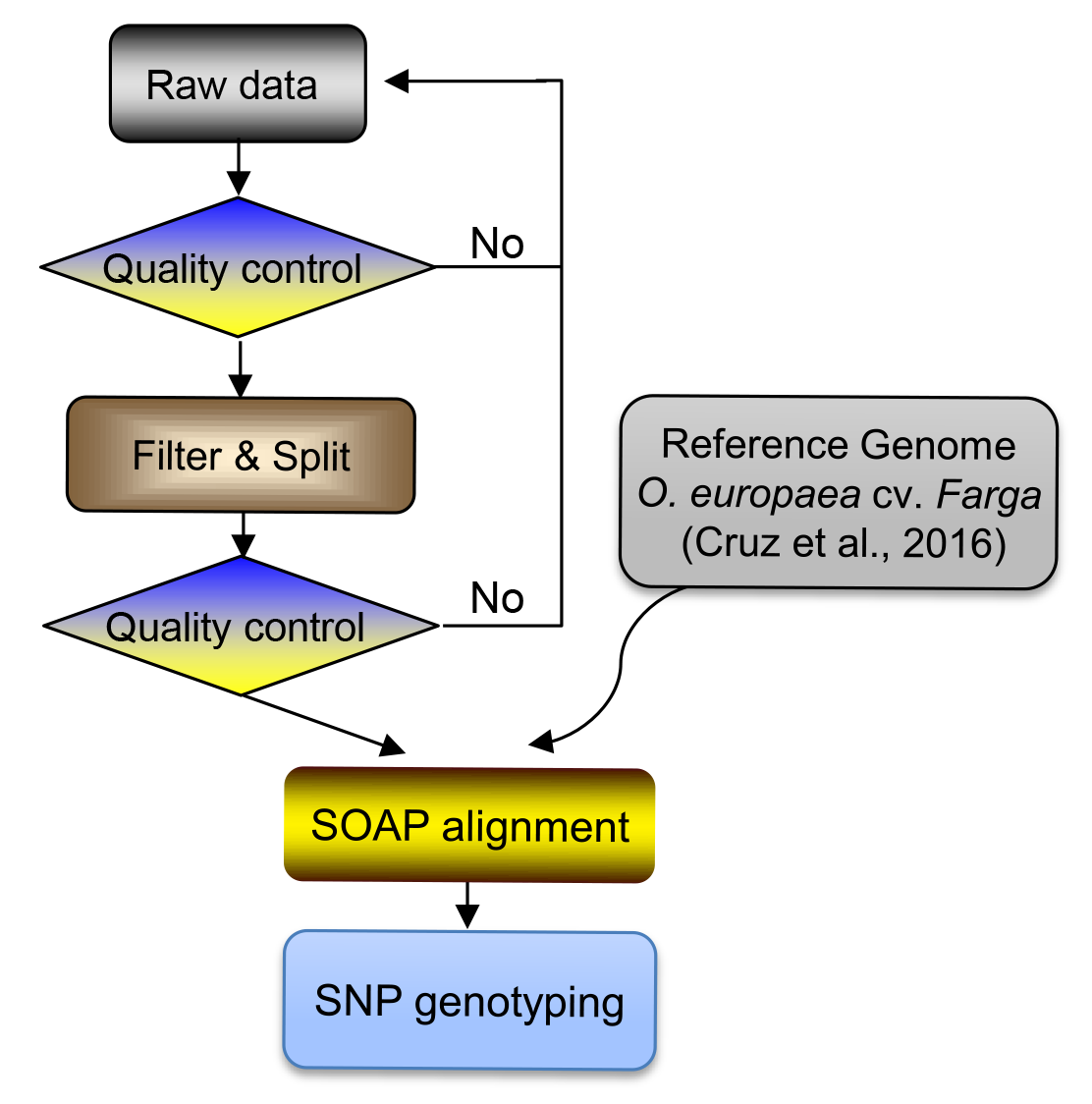

Supplement: Figure S1 — Bioinformatics analysis pipeline for GBS-SNP. The raw reads were subjected to quality control and split into clean reads. Using SOAP2 (Hurgobin, 2016) and SOAPsnp (Li et al., 2008), the clean reads were aligned to the olive reference sequences O. europaea cv. Farga (Cruz et al., 2016) and further to call SNP respectively. The main parameters were shown in Supplementary Table S2 . [file Image_1.tif]

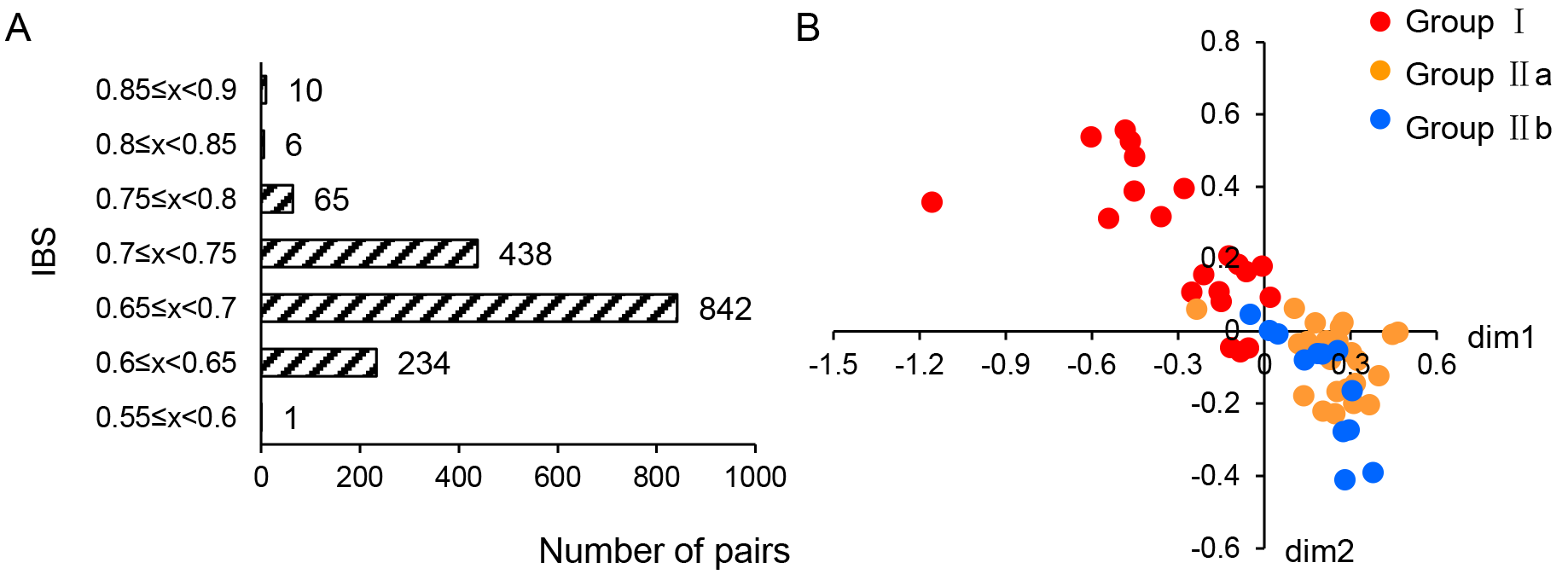

Supplement: Figure S2 — Multi-dimensional scaling (MDS) plot of identity-by-state (IBS) distance matrix. Pair-wise IBS allele-sharing using the 73,482 SNPs among 57 olive cultivars were calculated using PLINK96 V1.90 and visualized with the MDS plot (Purcell et al., 2007). [file Image_2.tif]
